# SHARE – workpackage 5: development of best practices of diagnosis and treatment for paediatric rheumatic diseases throughout Europe

**DOI:** 10.1186/1546-0096-12-S1-O7

**Published:** 2014-09-17

**Authors:** Sebastiaan Vastert, Pavla Dolezalova, Brian Feldman, Angelo Ravelli, Nico Wulffraat, Alberto Martini, Helene Foster, Felicitas Belluti Enders, Annet van Royen, Liza McCann, Noortje Groot, Sylvia Kamphuis, Tadej Avcin, Michael Beresford, Roberta Culpo, Ivan Foeldvari, Francesco Zulian

**Affiliations:** 1 Pediatric Rheumatology, University Medical Center Utrecht, Utrecht, Netherlands; 2 Paediatric Rheumatology Unit, 1st Faculty of Medicine, Prague, Czech Republic; 3 Pediatric Rheumatology, Hospital for Sick Children, Toronto, Canada; 4 Pediatric Rheumatology, G Gaslini Institute, Genova, Italy; 5 Department of Rheumatology, Newcastle, UK; 6 Pediatric Rheumatology, Universty Medical Center Utrecht, Netherlands; 7 Pediatric Rheumatology, Alder Hey Childrens’ NHS Foundation Trust, Liverpool, UK; 8 Pediatric Rheumatology, Erasmus Medical Center, Rotterdam, Netherlands; 9 Department of Pediatric Rheumatology, Ljubljana, Slovenia; 10 Department of Pediatric Rheumatology, University of Padua, Padua, Italy; 11 Hamburger Zentrum fuer Kinder- und Jugendrheumatologie, Hamburg, Germany

## Introduction

Paediatric rheumatic diseases (PRD) form a group of (rare) diseases that can lead to significant morbidity. Evidence-based guidelines are sparse and treatment regimens differ throughout Europe. In 2012, a European initiative called SHARE (Single Hub and Access point for pediatric Rheumatology in Europe) was granted by the European Agency for Health and Consumers (project number 2011 1202) to optimize and disseminate diagnostic and management regimens in Europe for children with PRD.

## Objectives

Workpackage 5 (WP5) aims to develop best practices of diagnosis and treatment for PRD.

## Methods

Evidence based recommendations were developed using the European League Against Rheumatism (EULAR) standard operating procedure [1]: Expert committees were formed, consisting of paediatric rheumatologists and experts in the 4 core PRD: Juvenile Idiopathic arthritis (JIA), Juvenile Dermatomyositis (JDM), Systemic Lupus Erythematosus (SLE, including antiphospholipid syndrome and vasculitis) and Juvenile Scleroderma (JSc, localized and systemic). The periodic fevers were incorporated in a later phase of the project and will be discussed separately. The expert committees defined domains and search terms for systematic literature reviews, which were executed in July 2013 in Medline, Embase and Cochrane databases. Subsequently, all available abstracts were checked for inclusion (published after 1970, English, no case reports, case series only when including at least 3 pediatric patients). All relevant papers were subsequently scored for validity and level of evidence (LOE) by 2 independent experts. In case of disagreement, a 3^rd^ independent expert confirmed the validity and LOE. Papers and scores were used to develop recommendations that were evaluated by all experts via an online survey as a 1^st^ step. Those with < 80% agreement in the survey were reformulated. Finally, recommendations were discussed at a consensus meeting with all experts present, using the nominal group technique [2]. Recommendations were accepted if > 80% agreement was reached.

## Results

Table 1 shows the number of scored papers after the standardised literature search and the number of recommendations on diagnosis and treatment for each PRD.

**Table 1:** 

Paediatric Rheumatic Disease	Nr of scored papers	Nr of recommendations
JIA	174	46

JDM	108	29

SLE/APS	143	36

Scleroderma	89	26

## Conclusion

Based upon standardised literature searches, WP5 of SHARE developed evidence based recommendations for diagnosis and treatment of PRD. These recommendations were discussed and agreed upon in a 1^st^ consensus meeting and will serve as input for the ultimate goal: development of best practices for care and management of PRD throughout Europe. These best practices will be finalized in a 2^nd^ consensus meeting in March 2015 and presented to all stakeholders including paediatric rheumatology units throughout Europe, patient/parent organisations and health authorities.

## Disclosure of interest

S. Vastert Consultant for: consultancy fees < 1000 euro in 2012 from Novartis, P. Dolezalova: None declared., B. Feldman: None declared, A. Ravelli: None declared, N. Wulffraat Grant / Research Support from: Abbvie, Roche, GSK, Consultant for: Novartis, Genzyme, Pfizer, Roche.
